# Short-chain fatty acids and intestinal inflammation in multiple sclerosis: modulation of female susceptibility by microbial products?

**DOI:** 10.1186/s13317-021-00149-1

**Published:** 2021-04-07

**Authors:** Anouck Becker, Mosab Abuazab, Andreas Schwiertz, Silke Walter, Klaus C. Faßbender, Mathias Fousse, Marcus M. Unger

**Affiliations:** 1 Department of Neurology, Saarland University, Kirrberger Str. 100, 66421 Homburg, Germany; 2 Klinik für Neurologie, Gesundheitszentrum Glantal, Liebfrauenberg 32, 55590 Meisenheim, Germany; 3 Institute of Microecology, Herborn, Germany; 4 Neuroscience Unit, Faculty of Medicine, Anglia Ruskin University, Chelmsford, Essex, UK

**Keywords:** Multiple Sclerosis, Intestinal inflammation, Short-chain fatty acids, Calprotectin, Female sex

## Abstract

**Background:**

Multiple Sclerosis (MS) is an autoimmune-mediated disease of the central nervous system. Experimental data suggest a role of intestinal microbiota and microbial products such as short-chain fatty acids (SCFAs) in the pathogenesis of MS. A recent clinical study reported beneficial effects (mediated by immunomodulatory mechanisms) after oral administration of the SCFA propionate in MS patients. Based on available evidence, we investigated whether SCFAs and the fecal inflammation marker calprotectin are altered in MS.

**Methods:**

76 subjects (41 patients with relapsing–remitting MS and 35 age-matched controls) were investigated in this case–control study. All subjects underwent clinical assessment with established clinical scales and provided fecal samples for a quantitative analysis of fecal SCFA and fecal calprotectin concentrations. Fecal markers were compared between MS patients and controls, and were analyzed for an association with demographic as well as clinical parameters.

**Results:**

Median fecal calprotectin concentrations were within normal range in both groups without any group-specific differences. Fecal SCFA concentrations showed a non-significant reduction in MS patients compared to healthy subjects. Female subjects showed significantly reduced SCFA concentrations compared to male subjects.

**Conclusions:**

In our cohort of MS patients, we found no evidence of an active intestinal inflammation. Yet, the vast majority of the investigated MS patients was under immunotherapy which might have affected the outcome measures. The sex-associated difference in fecal SCFA concentrations might at least partially explain female predominance in MS. Large-scale longitudinal studies including drug-naïve MS patients are required to determine the role of SCFAs in MS and to distinguish between disease-immanent effects and those caused by the therapeutic regime.

**Supplementary Information:**

The online version contains supplementary material available at 10.1186/s13317-021-00149-1.

## Background

Multiple Sclerosis (MS) is an auto-inflammatory disease of the central nervous system (CNS). Apart from trauma, it is the most common disease leading to disability in young adults worldwide [1]. MS can present with various neurological symptoms depending on the affected region in the CNS. There are three main subtypes of MS that are defined by the clinical course: relapsing–remitting MS (RRMS), primary progressive MS (PPMS) and secondary progressive MS (SPMS). Pathophysiologically, autoreactive Th1 and Th17 CD4^+^ T helper cells and a reduced frequency of regulatory T cells (Tregs) characterize the pro-inflammatory environment in MS [2, 3]. The experimental autoimmune encephalomyelitis (EAE) mouse model is the most widely used animal model for MS. In this model, mice receive CD4 + lymphocytes specific for myelin or become immunized with proteins or peptides deriving from myelin. This intervention results in CNS inflammation and MS-typical features [4]. EAE onset is strongly linked to microbial stimuli: colonization of germ-free mice with commensal bacteria leads to EAE development [5], while mice that are kept under germ-free condition do not routinely develop EAE. Short-chain fatty acids (SCFAs) are thought to be beneficial in EAE: the SCFA butyrate suppresses demyelination and enhance remyelination in mice via oligodendrocyte differentiation [6]. Other studies also reported significant beneficial effects in the EAE model by the SCFAs valerate and propionate [7, 13].

It is concluded that SCFAs, which are microbial products mainly produced by intestinal microbiota, counteract demyelination [6]. Hence, microbiota, microbial products and the intestinal immune system are likely to be relevant in the pathophysiology of MS. The SCFAs acetate, propionate and butyrate are most abundant in the gut. SCFAs are produced by intestinal microbiota via fermentation of dietary fibers. Acetate and propionate derive predominantly from members of the phylum *Bacteroidetes* (such as *Prevotellaceae*) while butyrate mainly originates from bacteria of the phylum *Firmicutes* (such as *Faecalibacterium*). Valerate is found in lower concentrations in the feces compared to the more abundant acetate, propionate and butyrate and is considered to derive from different dietary components [7].

Recently, a clinical trial reported a change in the composition of the intestinal microbiota accompanied by immunomodulatory effects (inter alia restoration of Treg frequency) and a beneficial clinical effect after oral propionate supplementation in drug-naïve MS patients [8].

In mouse models, SCFA showed pro- and anti-inflammatory effects [9, 10]. SCFA can enter the systemic circulation via the intestinal epithelium and cross the blood–brain barrier [3, 10]. SCFA interact with immune cells through in different mechanisms such as the NF-kB G-protein coupled receptors-mediated pathway. They lead to epigenetic modulations in T lymphocytes by inhibiting histone deacetylase activity [11], thus leading to higher frequencies of Tregs [12]. In turn, Tregs suppress overly active T-cell mediated immune responses. Valerate has been shown to strongly increase IL-10 levels in T- and in regulatory B-cells, a strong immunosuppressive mediator [7].

In summary, SCFAs, which derive from the intestinal microbiota, might be a modulating factor in MS pathology [13].

While a number of experimental data exist, only few studies investigated the intestinal microbiota and SCFAs in MS patients. Existing evidence in humans indicates that MS patients have an altered gut microbiota composition [15–17]. A Chinese study also reported a decrease in fecal SCFA concentrations [14] and a correlation of fecal SCFA concentrations with Treg frequency in MS patients. Another study reported reduced SCFA blood concentrations in patients with secondary progressive MS [15].

Fecal calprotectin (a protein derived from leukocytes that migrate into the intestinal lumen under inflammatory conditions) is a stable and sensitive established marker for inflammatory activity in Crohn’s disease and other inflammatory bowel disease (IBD) [16]. Elevated fecal calprotectin concentrations have not only been described in IBD, but also in other neurological disorders such as Parkinson’s disease [17, 18].

Considering the plausible interaction between gut microbiota, host immunity and microbial products and considering the recent advances in research of the gut-brain-axis, the limited amount of clinical evidence in humans prompted us to investigate fecal calprotectin and fecal SCFAs concentrations in RRMS patients and age-matched healthy controls in a case–control study.

## Subjects and methods

This case–control study was reviewed and approved by the local ethics committee (Ethikkommission der Ärztekammer des Saarlandes, registration number 81/18). All subjects provided written informed consent.

Subjects were assessed between 2018 and 2019 at the Department of Neurology of the Saarland University Medical Center, Germany and the Gesundheitszentrum Glantal, Germany. Inclusion criteria for patients were a diagnosis of a RRMS according to Lublin’s consensus classification of 2013 [19] and ability to provide written informed consent. Exclusion criteria for patients and controls were pregnancy, lack of legal capacity, uncontrolled psychiatric diseases, neurodegenerative disorders, any disease of acute or chronic intestinal inflammation, a coexistent infection within the past four weeks and intake of antibiotics during the past eight weeks. The presence of a MS relapse at the time of investigation was also an exclusion criterion. For control subjects, presence or history of any autoimmune-mediated disease was an additional exclusion criterion. Patients were grouped in mild / moderately active disease and active/highly active disease (formerly: “aggressive MS”) due to clinical criteria such as EDSS, MRI disease activity, treatment subgroup and response to disease modifying drugs [20, 21].

For analysis of different treatment subgroups, betaferones, glatirameracetate, teriflunomid and dimethylfumarate were defined as *basic therapy*, whereas natalizumab and fingolimod were defined as *escalation therapy*. This is based on the use of the basic therapy drugs as first line therapy in mild / moderate MS whereas the escalation therapy is usually second line (although it can also be used as first line in highly active MS depending on local drug approval for this clinical situation) [22, 23].

All subjects underwent a structured medical history and a clinical examination including scoring with the *Expanded Disability Status Scale* (EDSS) [24], *Mini-mental status test *[25], *Fatigue Impact Scale (FIS) *[26] and the *Beck Depression Inventory (BDI) *[27]. All subjects were provided with a fecal sampling kit and instructions on how to collect fecal samples at home as reported previously [28]. Fecal SCFA and fecal calprotectin concentrations were quantitatively analyzed as previously described [28]. Blood C-reactive protein (CRP) concentrations were available for the majority of subjects, but were not explicitly part of the study protocol.

Data analysis was carried out with IBM SPSS, version 24®. Normal distribution of data was tested using the Shapiro–Wilk test. Except for FIS- and BDI-scores, data was not normally distributed. Hence, results are reported as median plus range (minimum to maximum). Mann–Whitney-U and Kruskal–Wallis test were used to compare both groups. Correlation between metric variables was analyzed using the Pearson’s correlation coefficient, Spearman’s correlation coefficient was used to analyze correlations between metric and ordinally scaled parameters. Eta correlation coefficient was used when analyzing correlations of metric and nominal variables. Statistical significance was assumed for p < 0.05 (with a statistical power of 0.8).

## Results

### Demographic and clinical data

RRMS patients (n = 41) and controls (n = 35) were matched for age (Table 1). All subjects were of Caucasian ethnicity. There was a female predominance in the RRMS group (29 of 41 subjects). EDSS scores were significantly higher in RRMS patients with an active / highly active RRMS (n = 23, median: 3, range 1 to 7) compared to those with a mild / moderate disease activity (n = 18, median: 2.5, range 0 to 7, p 0.004). All but three RRMS patients were under immunotherapies. Detailed information concerning individual treatment and disease activity is provided in Additional file 1: Table S1. None of the subjects enrolled in this case–control study showed a clinically relevant increase in CRP concentration (data available for 29 of 35 control subjects and 27 of 41 RRMS patients, Table 1). None of the enrolled subjects was currently under corticosteroid therapy; the latency to the last corticosteroid therapy (if any) has not been included in the study protocol.

**Table 1:** Distribution of epidemiological and clinical data among subjects

	MS patients (n = 41)	Controls (n = 35)	All subjects (n = 76)
Age in years median [range]	48 [22–68]	48 [23–72]	48 [22–72]
Sex f:m	29:12	13:22	42:34
EDSS score median [range]	2.5 [0–7.0]	Not applicable	Not applicable
CRP in mg/l median [range]	1.3 [1.0–9.6]	1.1 [1.0–14.0]	1.3 [1.0–14.0]

### Fecal calprotectin concentrations

**Fig. 1:**
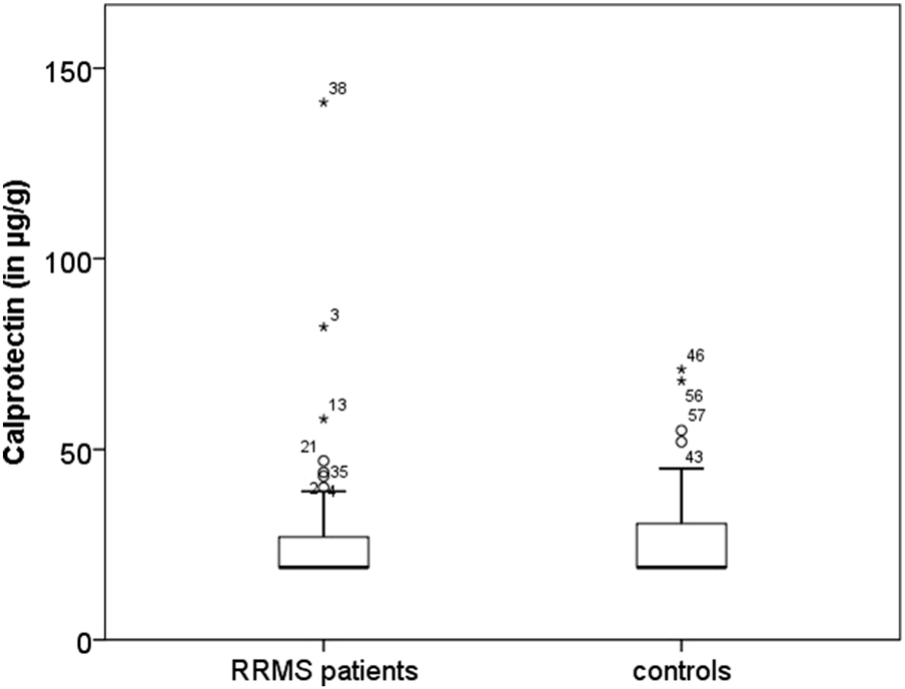
Fecal calprotectin in patients and controls visualized as boxplot. The control group contains an outlier with a fecal calprotectin concentration of 328 µg/g, not depicted for better visualization

No significant difference existed between the fecal calprotectin concentrations of RRMS patients (median: 19 µg/g, range 19–141 µg/g) and healthy controls (median: 19 µg/g, range 19–328 µg/g) as shown in Fig. 1. Concentration were within normal range in both groups. There was no difference in fecal calprotectin concentrations between RRMS patients under basic therapy compared to those under escalation therapy, between mild disease compared to (highly) active disease, between patients with low or high EDSS and no difference between different drugs (data not shown). No significant difference in fecal calprotectin concentrations between both sexes was observed.

### Fecal short-chain fatty acid concentrations

**Fig. 2:**
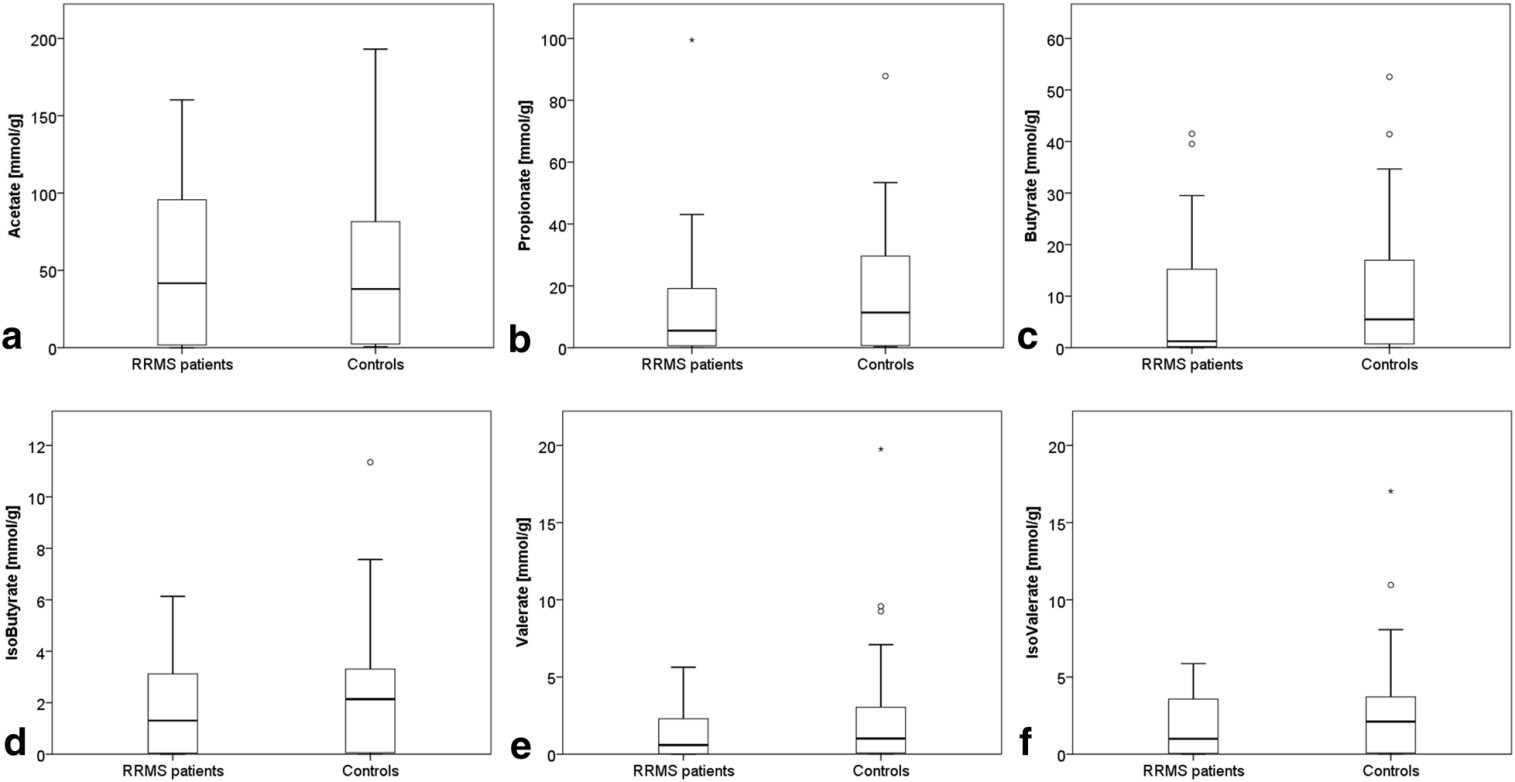
Fecal SCFA concentrations in patients and controls

**Fig. 3:**
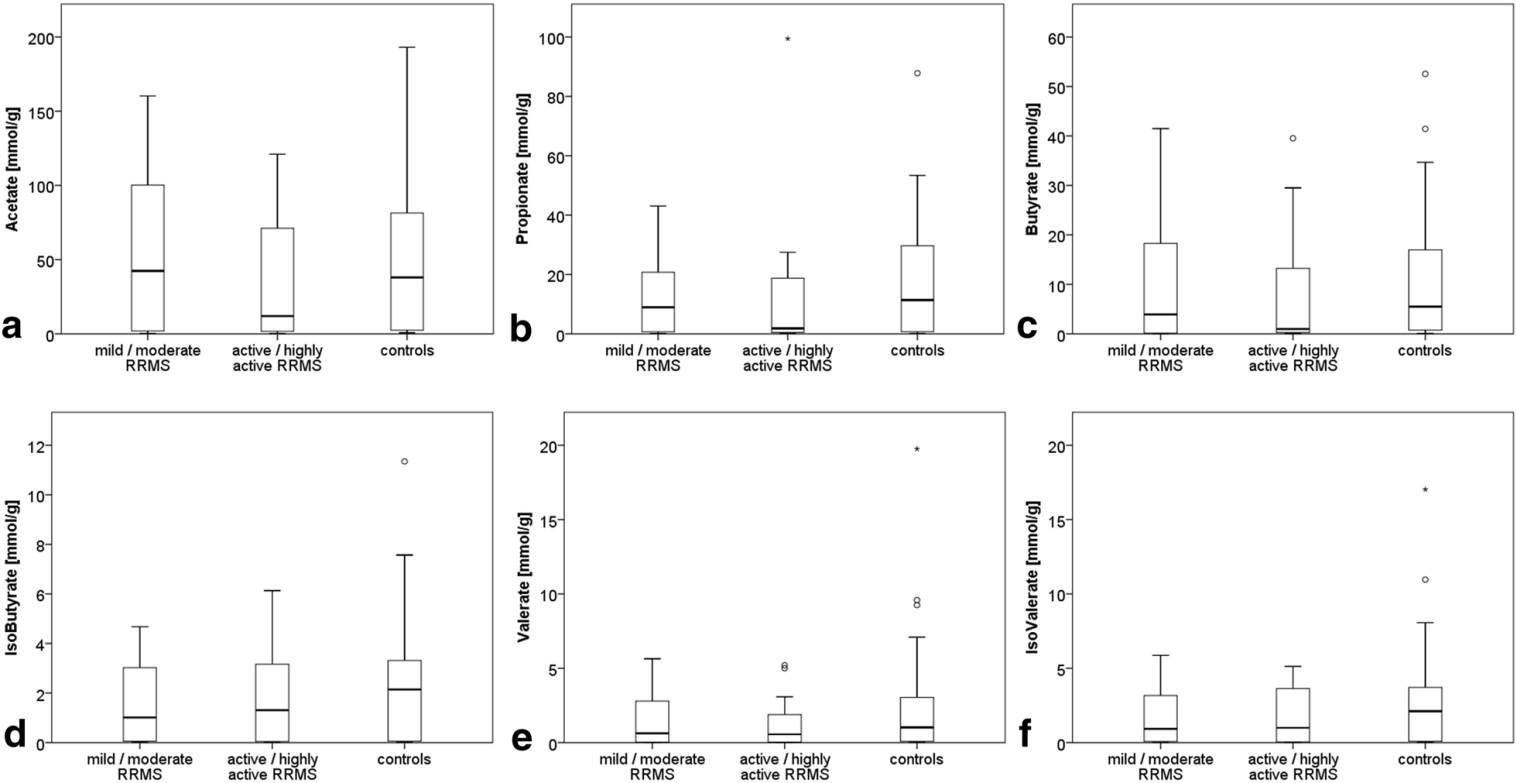
Fecal SCFA concentrations sorted for disease activity

SCFA concentrations were descriptively lower in RRMS patients compared to controls (e.g. median fecal butyrate concentrations were reduced by 77% in RRMS patients compared to control subjects). Yet, there was no statistically significant difference in fecal SCFA concentrations between RRMS patients and controls (Table 2, Fig. 2). Median fecal acetate concentration was reduced by 72% in patients with an active/highly active RRMS compared to those with a mild/moderate activity; this finding was also not statistically significant (p 0.554) (Fig. 3). EDSS scores did not correlate with fecal SCFA concentrations (data not shown).

**Table 2:** Shows median fecal SCFA-concentrations (in mmol/g) in patients and controls

SCFA	Patients (n = 41)	Controls (n = 35)	p
Acetate in mmol/g median [range]	41.70 [0.07–160.26]	37.95 [0.66–193.06]	0.606
Propionate in mmol/g median [range]	5.51 [0.10–99.47]	11.38 [0.17–87.81]	0.258
Butyrate in mmol/g median [range]	1.25 [0.02–41.49]	5.49 [0.05–52.54]	0.219
IsoButyrate in mmol/g median [range]	1.31 [0.00–6.13]	2.14 [0.01–11.35]	0.171
Valerate in mmol/g median [range]	0.60 [0.00–5.64]	1.02 [0.01–19.76]	0.072
IsoValerate in mmol/g median [range]	1.00 [0.01–5.87]	2.11 [0.01–17.03]	0.128

**Fig. 4:**
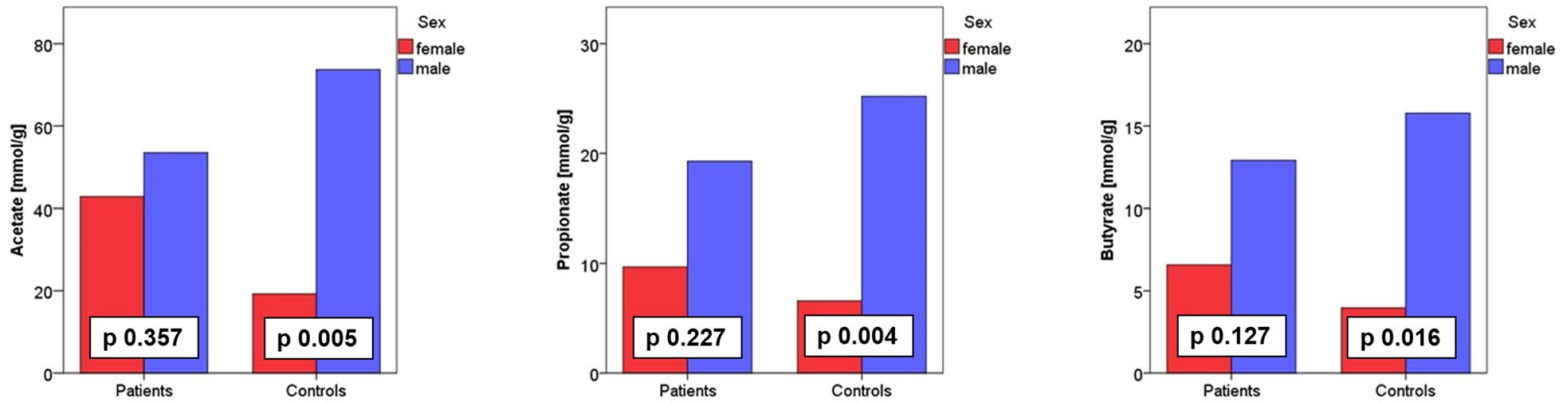
Fecal acetate concentration in female and male subjects visualized as bar chart

Aside from the branched SCFAs iso-valerate and iso-butyrate, all SCFAs concentrations were significantly lower in women compared to men (Table 3, Fig. 4). Analyzing RRMS patients and controls separately, acetate, propionate and butyrate were significantly lower in women compared to men in the control group (Table 3, Fig. 4); for RRMS patients, there was also a descriptive difference. Yet, in RRMS this difference did not reach statistical significance (Table 3, Fig. 4).

**Table 3:** Shows fecal SCFA concentrations (in mmol/g, median and range) and the respective p value (difference between female and male subjects) for each investigated SCFA separately

	Patients (n = 41)			Controls (n = 35)			All (n = 76)		
	m n = 12	f n = 29	p	m n = 22	f n = 13	p	m n = 34	f n = 42	p
Acetate mmol/g	51.48 [0.67–121.05]	3.76 [0.07–160.26]	0.357	73.4 [1.04–193.06]	8.02 [0.66–71.22]	**0.005**	68.89 [0.67–193.06]	6.47 [0.07–160.26]	**0.012**
Propionate mmol/g	14.25 [0.37–99.47]	1.29 [0.1–43.07]	0.227	21.09 [0.22–87.81]	1.59 [0.17–27.08]	**0.004**	19.40 [0.22–99.47]	1.44 [0.01–43.07]	**0.002**
Butyrate mmol/g	9.17 [0.14–39.53]	0.88 [0.02–41.49]	0.127	15.95 [0.05–52.54]	1.89 [0.08–18.98]	0.16	15.63 [0.55–52.54]	1.37 [0.02–41.49]	**0.003**
IsoButyrate mmol/g	1.9 [0.02–4.23]	0.97 [0.004–6.13]	0.436	2.33 [0.02–11.35]	1.55 [0.01–4.01]	0.243	2.10 [0.02–11.35]	0.88 [0.00–6.13]	0.061
Valerate mmol/g	1.55 [0.01–5.2]	0.96 [0.002–5.64]	0.142	2.07 [0.01–19.76]	0.63 [0.01–3.05]	0.067	1.58 [0.01–19.76]	0.53 [0.00–5.64]	**0.004**
Isovalerate mmol/g	1.6 [0.02–5.78]	0.099 [0.007–5.49]	0.342	2.25 [0.02–17.03]	2.04 [0.01–5.32]	0.335	1.94 [0.02–17.03]	0.55 [0.01–5.49]	0.068

All fecal SCFA concentrations showed a statistically significant negative correlation with age when analyzing all subjects included in the study or analyzing control subjects; RRMS patients did not show a significant correlation of SCFAs with age (Table 4).

**Table 4:** Shows the Pearson’s correlation coefficient and respective p values for the correlation between age and fecal SCFA concentrations

	Patients (n = 41)		Controls (n = 35)		All subjects (n = 76)	
	Pearson’s correlation coefficient	p	Pearson’s correlation coefficient	p	Pearson’s correlation coefficient	p
Acetate	− 0.128	0.435	− 0.522	0.001	− 0.335	0.003
Propionate	− 0.026	0.874	− 0.483	0.003	− 0.268	0.019
Butyrate	− 0.297	0.297	− 0.480	0.003	− 0.335	0.003
IsoButyrate	− 0.043	0.791	− 0.452	0.006	− 0.279	0.015
Valerate	− 0.088	0.584	− 0.444	0.006	− 0.316	0.005
Isovalerate	− 0.01	0.952	− 0.434	0.009	− 0.275	0.016

## Discussion

Experimental studies suggest that microbiota, microbial products (like SCFAs) and the intestinal immune system might be involved in the pathogenesis of MS. Hitherto, only sparse clinical data exist. In this case–control study we investigated fecal markers of intestinal inflammation in RRMS patients and age-matched control subjects. In consideration of the current evidence pointing at a disturbed gut microbiome, we hypothesized that MS patients show increased markers of intestinal inflammation (investigated by the surrogate marker fecal calprotectin) and reduced concentrations of SCFAs. While there were no elevated calprotectin concentrations and only a descriptive reduction of SCFA concentrations in RRMS patients, we observed a significant sex-related difference of fecal SCFA concentrations between men and women as a possible indicator of MS susceptibility differences among the sexes.

Contrary to what we initially hypothesized, fecal calprotectin concentrations, a robust and sensitive marker even for subclinical intestinal inflammation, was in the normal range in the majority of investigated RRMS patients and there was no difference regarding fecal calprotectin concentrations between RRMS and control subjects. While there is one study reporting elevated calprotectin concentrations in the cerebrospinal fluid of MS patients [29], fecal calprotectin concentrations have not been reported for MS previously. The fact that most investigated RRMS patients were under immunotherapy, which beside their effect on the CNS alters enteric inflammatory processes as well, might explain the finding of normal fecal calprotectin concentrations in our RRMS cohort. Consequently, the observation of normal fecal calprotectin concentrations in our RRMS cohort might be particularly explained by the fact that 14 of the investigated 41 RRMS patients were treated with natalizumab, a drug also used in the treatment of Crohn’s disease [30].

Assuming that immunotherapies in MS exert anti-inflammatory effects also in the gastrointestinal tract, the intestinal microbiota (as indicated by Storm-Larsen et al. for dimethylfumarate [27]) and subsequently intestinal SCFA production might be affected as well. Hence, the lack of a significant difference between RRMS patients and controls with regard to fecal SCFA concentrations in this study might also be explained by a drug effect.

Despite the lack of a statistical significance, we observed descriptively lower fecal SCFA concentrations in RRMS patients compared to control subjects, especially for butyrate. This descriptive finding is in line with the few studies investigating SCFA in MS: Park et al. showed, that SCFA blood concentrations were reduced in MS patients [15]. Yet, blood SCFA concentrations are not directly comparable with the fecal concentrations of SCFAs we investigated in this study. A Chinese study reported reduced fecal SCFA concentrations in MS patients [14]. An altered intestinal microbiota has been reported in MS patients as well [31–33]. Moreover, the highly significant correlation of fecal SCFA concentrations with age in controls, but not in patients, endorses the assumption that either MS or MS therapeutics affect the gut microbiome.

Recently, the potential clinical relevance of SCFAs in MS has been investigated in a clinical trial [8]: Duscha et al. reported an enhancement of Treg differentiation, reduced auto-inflammation and improvements in the clinical course of MS after oral administration of propionate [8]. It is important to note that orally administered SCFA are absorbed to a great extent in the small intestine. SCFA produced by the gut microbiota in the colon mainly exert local effects and are unlikely to affect systemic SCFA concentrations as effective as an oral supplementation.

We are not able to draw conclusions concerning fecal calprotectin and SCFA concentrations in drug-naïve MS patients as the vast majority of RRMS patients in this study was under immunotherapy. Diet was not explicitly investigated as part of the study protocol, so dietary habits are a potential confounding factor. As the investigated RRMS patients were under different treatment regimes, we also analyzed subgroups of RRMS patients defined by the therapeutic regime. Yet, the number of subjects per subgroup was rather small and the study population was not treated with the full spectrum of available MS therapies. Large-scale longitudinal studies, including drug-naïve MS patients are necessary to distinguish between disease-immanent and therapeutic effects on intestinal inflammation, intestinal microbiota and microbial products, like SCFA, in MS. Another interesting topic for future investigations is the role of (subclinical) intestinal inflammation as a trigger for relapse in MS.

An unexpected finding of our study was the marked sex-associated difference in SCFA concentrations between women and men with significantly lower SCFA concentrations in female subjects. Sex-specific differences have been described for the intestinal microbiota previously [34]. Fecal SCFA concentrations have already been subject of clinical studies in different fields, e.g. anorexia [29], obesity, diabetes mellitus and cardiometabolic disease [30]. Yet, none of these studies reported sex-specific differences for fecal SCFA concentrations. It might well be that this aspect was not explicitly analyzed in these studies.

Jakobsdottir and colleagues reported sex-specific differences of blood SCFA concentrations (with lower SCFA concentrations in female subjects) in a study comparing patients with microscopic colitis and celiac disease [35]. Another study did not find sex-specific differences when analyzing blood SCFA concentrations [36]. As already mentioned, blood and fecal SCFA concentrations are not directly comparable.

RRMS patients and control subjects in this study were matched for age, but there was a male predominance in the control group, which represents a potential confounding factor.

Taken together, the known female predominance in MS and the known immunomodulatory effects of SCFAs warrant further studies in this field. One might hypothesize that low concentrations of SCFA represent an additional risk factor for MS and might contribute to the higher susceptibility of women compared to men in MS. As the observed sex-specific difference in SCFA concentrations was independent from MS, also studies in other conditions that investigate microbiota and microbial products should consider sex as a potential confounding factor.

## Supplementary Information

**Additional file 1: Table S1.** Synopsis of all enrolled subjects. 1 Age at enrollment in years. 2 Sex. m=male, f=female. 3 disease activity: mi/mod=mild/moderate, act/ha=active/highly active. 4 Drug: Nat=Natalizumab, Dmf=dimethylfumarate, Glat=glatirameracetate, ßIFN=betaferones, PIF=pegylated betaferones, Fin=fingolimod, /=none. 5 c-reactive protein in blood in mg/l. *= data not available. 6 fecal calprotectin in µg/g. 7–12 fecal concentration of acetate(Acet)/propionate(Prop)/butyrate(But)/isobutyrate(Isobut)/valerate(Val)/isovalerate(Isoval) in mmol/g.

## Data Availability

The datasets supporting the conclusions of this article are included within the article and its Additional file 1.

## Abbreviations

CNS: Central nervous system
EAE: Experimental autoimmune encephalomyelitis
MS: Multiple sclerosis
PPMS: Primary progressive multiple sclerosis
RRMS: Relapsing remitting multiple sclerosis
SCFA: Short-chain fatty acids
SD: Standard deviation
SPMS: Secondary progressive multiple sclerosis
ST: Student’s t-test (unpaired)
Tregs: Regulatory T cells
