# Supplementary material for: Short-chain fatty acids and intestinal inflammation in multiple sclerosis: modulation of female susceptibility by microbial products?
Source: Auto Immun Highlights. 2021 Apr 7;12(1):7. doi: 10.1186/s13317-021-00149-1 (PMC8028206; doi:10.1186/s13317-021-00149-1)
Supplement: Supplementary file 1 — Additional file 1: Table S1. Synopsis of all enrolled subjects. 1 Age at enrollment in years. 2 Sex. m=male, f=female. 3 disease activity: mi/mod=mild/moderate, act/ha=active/highly active. 4 Drug: Nat=Natalizumab, Dmf=dimethylfumarate, Glat=glatirameracetate, ßIFN=betaferones, PIF=pegylated betaferones, Fin=fingolimod, /=none. 5 c-reactive protein in blood in mg/l. *= data not available. 6 fecal calprotectin in µg/g. 7–12 fecal concentration of acetate(Acet)/propionate(Prop)/butyrate(But)/isobutyrate(Isobut)/valerate(Val)/isovalerate(Isoval) in mmol/g. [file 13317_2021_149_MOESM1_ESM.docx]

Table S1 Synopsis of all enrolled subjects. **1** Age at enrollment in years. **2** Sex. m=male, f=female. **3** disease activity: mi/mod=mild/moderate, act/ha=active/highly active. **4** Drug: Nat=Natalizumab, Dmf=dimethylfumarate, Glat=glatirameracetate, ßIFN=betaferones, PIF=pegylated betaferones, Fin=fingolimod, /=none. **5** c-reactive protein in blood in mg/l. *=data not available. **6** fecal calprotectin in µg/g. **7-12** fecal concentration of acetate(Acet)/propionate(Prop)/butyrate(But)/isobutyrate(Isobut)/valerate(Val)/isovalerate(Isoval) in mmol/g.

| **Patient** | **Age^1^** | **Sex^2^** | **disease activity^3^** | **Drug^4^** | **EDSS**  **score** | **CRP^5^**  **mg/l** | **Calp.^6^**  **µg/g** | **Acet^7^**  **mmol/g** | **Prop^8^**  **mmol/g** | **But^9^**  **mmol/g** | **Isobut^10^**  **mmol/g** | **Val^11^**  **mmol/g** | **Isoval^12^**  **mmol/g** | |
| --- | --- | --- | --- | --- | --- | --- | --- | --- | --- | --- | --- | --- | --- | --- |
| 1 | 28 | f | act/ha | Nat | 2.0 | 1.3 | 22.0 | 1.441 | .435 | .295 | .048 | .041 | .053 | |
| 2 | 25 | f | mi/mod | Dmf | .0 | 1.2 | 44.0 | 95.665 | 17.464 | 18.255 | 3.023 | 2.796 | 3.170 | |
| 3 | 36 | f | act/ha | Nat | 2.0 | 6.0 | 82.0 | 2.281 | .814 | .827 | .013 | .040 | .015 | |
| 4 | 59 | f | act/ha | Nat | 3.5 | 1.3 | 43.0 | 2.060 | 1.078 | .678 | .039 | .080 | .053 | |
| 5 | 64 | f | act/ha | Nat | 3.5 | 3.4 | 19.0 | .069 | .100 | .020 | .008 | .002 | .010 | |
| 6 | 62 | f | mi/mod | / | 4.0 | * | 19.0 | 3.764 | 1.288 | 1.250 | .074 | .072 | .089 | |
| 7 | 40 | f | act/ha | Nat | 3.0 | 1.0 | 19.0 | .463 | .188 | .088 | .013 | .005 | .020 | |
| 8 | 47 | f | mi/mod | Dmf | 2.0 | 3.5 | 19.0 | 1.856 | .617 | .144 | .059 | .023 | .070 | |
| 9 | 53 | f | mi/mod | Glat | 1.5 | * | 27.0 | 1.679 | .928 | .145 | .054 | .006 | .075 | |
| 10 | 40 | f | act/ha | Nat | 2.0 | 1.0 | 19.0 | 1.703 | .596 | .200 | .046 | .027 | .053 | |
| 11 | 31 | f | act/ha | Nat | 1.0 | 9.6 | 19.0 | 1.894 | .490 | .365 | .058 | .042 | .068 | |
| 12 | 27 | f | act/ha | Nat | 3.0 | 1.0 | 19.0 | 2.324 | .462 | .214 | .070 | .002 | .099 | |
| 13 | 50 | f | mi/mod | / | 1.5 | * | 58.0 | 1.395 | .550 | .118 | .056 | .028 | .078 | |
| 14 | 68 | f | act/ha | Nat | 3.0 | 1.7 | 19.0 | .931 | .183 | .054 | .017 | .018 | .031 | |
| 15 | 66 | f | mi/mod | PIF | 2.0 | 1.0 | 19.0 | 1.983 | .764 | .341 | .030 | .010 | .043 | |
| 16 | 45 | f | mi/mod | βIFN | 2.5 | * | 19.0 | .286 | .139 | .035 | .004 | .011 | .007 | |
| 17 | 31 | f | act/ha | PIF | 3.5 | * | 19.0 | 52.655 | 9.841 | 6.486 | 3.838 | 2.279 | 4.235 | |
| 18 | 34 | f | mi/mod | Dmf | 1.5 | * | 19.0 | 80.296 | 20.748 | 6.589 | 2.339 | .595 | 1.891 | |
| 19 | 68 | f | mi/mod | Glat | .0 | 1.0 | 23.0 | 103.538 | 17.657 | 9.705 | 4.325 | 3.622 | 4.556 | |
| 20 | 44 | f | mi/mod | Glat | 1.5 | * | 19.0 | 100.184 | 31.042 | 41.494 | .674 | 2.736 | .725 | |
| 21 | 47 | f | act/ha | Nat | 7.0 | 2.4 | 47.0 | 110.934 | 26.482 | 11.800 | 1.306 | .652 | .995 | |
| 22 | 52 | f | act/ha | Nat | 3.5 | 1.0 | 34.0 | 48.980 | 5.510 | 1.877 | 2.255 | 1.008 | 2.233 | |
| 23 | 56 | f | act/ha | Fin | 3.5 | 1.5 | 19.0 | 55.233 | 16.785 | 4.713 | 3.249 | 1.938 | 3.703 | |
| 24 | 57 | f | mi/mod | Glat | .0 | 1.1 | 19.0 | 112.146 | 24.916 | 18.310 | 4.677 | 4.141 | 5.494 | |
| 25 | 50 | f | act/ha | Nat | 3.5 | 1.0 | 19.0 | 96.381 | 19.142 | 15.198 | 6.134 | .620 | 5.124 | |
| 26 | 58 | f | mi/mod | Glat | 2.0 | * | 39.0 | 41.696 | 7.721 | .881 | 1.353 | .659 | 2.016 | |
| 27 | 37 | f | mi/mod | Dmf | 2.5 | 3.8 | 19.0 | 160.261 | 43.072 | 12.159 | 4.355 | 5.636 | 4.358 | |
| 28 | 56 | f | act/ha | Fin | 1.5 | 3.5 | 19.0 | 64.276 | 12.711 | 14.688 | 2.607 | 1.827 | 2.630 | |
| 29 | 48 | f | mi/mod | Glat | 4.5 | * | 25.0 | 95.239 | 19.123 | 23.803 | 1.392 | 2.297 | 1.128 | |
| 30 | 37 | m | act/ha | Nat | 5.0 | 1.0 | 19.0 | 1.679 | .770 | .355 | .018 | .027 | .017 | |
| 31 | 47 | m | mi/mod | Dmf | 1.5 | 6.3 | 19.0 | 2.452 | .671 | .398 | .029 | .013 | .031 | |
| 32 | 54 | m | act/ha | Dmf | 2.5 | * | 19.0 | 1.492 | .405 | .165 | .056 | .054 | .082 | |
| 33 | 60 | m | mi/mod | Glat | 3.0 | * | 25.0 | .667 | .373 | .141 | .015 | .028 | .019 | |
| 34 | 47 | m | act/ha | Fin | 5.0 | 1.6 | 19.0 | 59.916 | 18.336 | 2.735 | 3.452 | 1.709 | 4.823 | |
| 35 | 50 | m | act/ha | Fin | 2.0 | 1.0 | 40.0 | 77.957 | 27.491 | 17.205 | 3.122 | 3.079 | 3.573 | |
| 36 | 41 | m | mi/mod | / | .0 | * | 39.0 | 106.803 | 21.205 | 21.706 | 1.683 | 1.388 | 1.266 | |
| 37 | 22 | m | act/ha | Nat | 1.0 | 1.0 | 19.0 | 121.052 | 26.487 | 39.531 | 4.231 | 5.198 | 4.789 | |
| 38 | 48 | m | act/ha | βIF | 3.0 | 1.5 | 141.0 | 114.128 | 24.238 | 29.497 | 3.198 | 2.908 | 4.071 | |
| 39 | 59 | m | mi/mod | PIF | 2.0 | * | 19.0 | 43.034 | 10.154 | 15.601 | 4.178 | 3.130 | 5.869 | |
| 40 | 54 | m | act/ha | Fin | 3.0 | * | 19.0 | 100.565 | 99.466 | 26.665 | 2.058 | 5.003 | 1.697 | |
| 41 | 37 | m | act/ha | Fin | 3.5 | 2.0 | 19.0 | 11.970 | 1.823 | .963 | 1.738 | .553 | 1.504 | |
| **Control** | | | | | | | | | | | | | |  |
| 1 | 72 | f |  | | | 5.8 | 19.0 | .703 | .294 | .076 | .023 | .020 | .039 | |
| 2 | 31 | f |  |  |  | 1.0 | 52.0 | .680 | .173 | .094 | .023 | .024 | .037 | |
| 3 | 60 | f |  |  |  | 1.6 | 19.0 | .660 | .195 | .101 | .008 | .013 | .012 | |
| 4 | 59 | f |  |  |  | 3.7 | 19.0 | 1.333 | .343 | .141 | .053 | .042 | .084 | |
| 5 | 47 | f |  |  |  | 1.0 | 71.0 | 2.036 | .650 | .510 | .055 | .084 | .065 | |
| 6 | 56 | f |  |  |  | 6.9 | 29.0 | 47.081 | 10.189 | 5.493 | 3.229 | 1.599 | 4.037 | |
| 7 | 48 | f |  |  |  | 1.0 | 19.0 | 37.953 | 11.948 | 6.394 | 3.633 | 2.005 | 4.619 | |
| 8 | 23 | f |  |  |  | * | 19.0 | 31.064 | 16.193 | 9.861 | 2.954 | 1.973 | 2.886 | |
| 9 | 29 | f |  |  |  | 1.4 | 19.0 | 4.925 | .591 | 1.497 | 2.213 | 1.017 | 2.114 | |
| 10 | 60 | f |  |  |  | 1.0 | 32.0 | 8.015 | 1.586 | 1.887 | 1.089 | .472 | .384 | |
| 11 | 56 | f |  |  |  | 1.0 | 19.0 | 71.217 | 27.081 | 18.977 | 4.008 | 3.052 | 5.319 | |
| 12 | 58 | f |  |  |  | 1.8 | 39.0 | 11.980 | 9.136 | 3.045 | 1.549 | .628 | 2.042 | |
| 13 | 36 | f |  |  |  | 1.0 | 19.0 | 32.454 | 7.169 | 3.467 | 2.324 | 1.344 | 2.552 | |
| 14 | 50 | m |  |  |  | 1.4 | 19.0 | 1.192 | .224 | .053 | .019 | .011 | .020 | |
| 15 | 43 | m |  |  |  | 1.0 | 68.0 | 2.799 | 1.106 | .813 | .068 | .078 | .085 | |
| 16 | 47 | m |  |  |  | 2.6 | 55.0 | 1.185 | .413 | .290 | .044 | .046 | .059 | |
| 17 | 58 | m |  |  |  | * | 19.0 | 1.630 | .736 | .670 | .063 | .097 | .083 | |
| 18 | 58 | m |  |  |  | 1.0 | 45.0 | 1.040 | .389 | .153 | .039 | .042 | .061 | |
| 19 | 57 | m |  |  |  | 6.0 | 19.0 | 4.068 | .738 | 1.572 | .051 | .073 | .047 | |
| 20 | 56 | m |  |  |  | 1.0 | 19.0 | 45.773 | 11.383 | 4.524 | 2.516 | 1.456 | 2.730 | |
| 21 | 61 | m |  |  |  | 2.7 | 25.0 | 32.743 | 11.241 | 1.889 | 2.141 | .873 | 2.316 | |
| 22 | 67 | m |  |  |  | 1.0 | 19.0 | 77.196 | 14.998 | 14.890 | 2.652 | 3.031 | 2.191 | |
| 23 | 30 | m |  |  |  | 14.0 | 19.0 | 81.722 | 34.575 | 16.242 | 4.270 | 9.585 | 6.453 | |
| 24 | 27 | m |  |  |  | * | 19.0 | 148.074 | 53.377 | 27.238 | .972 | .836 | 1.117 | |
| 25 | 53 | m |  |  |  | 1.0 | 19.0 | 68.164 | 19.806 | 31.639 | 1.876 | 2.679 | 1.477 | |
| 26 | 30 | m |  |  |  | 11.0 | 328.0 | 164.660 | 35.097 | 16.843 | 7.570 | 6.708 | 10.961 | |
| 27 | 40 | m |  |  |  | * | 27.0 | 69.609 | 18.997 | 16.251 | 2.667 | 2.791 | 2.394 | |
| 28 | 39 | m |  |  |  | * | 19.0 | 96.990 | 29.306 | 17.802 | 3.395 | .870 | 3.388 | |
| 29 | 27 | m |  |  |  | 2.2 | 19.0 | 117.518 | 45.650 | 17.083 | 1.772 | 3.239 | 1.325 | |
| 30 | 48 | m |  |  |  | 1.0 | 33.0 | 94.564 | 33.450 | 27.484 | 2.858 | .556 | 2.409 | |
| 31 | 61 | m |  |  |  | 1.1 | 19.0 | 81.134 | 50.272 | 7.439 | 1.815 | 3.944 | 1.667 | |
| 32 | 29 | m |  |  |  | 1.0 | 19.0 | 193.064 | 87.805 | 34.681 | 6.323 | 19.759 | 7.551 | |
| 33 | 29 | m |  |  |  | 1.0 | 19.0 | 135.236 | 30.003 | 52.543 | 11.349 | 9.252 | 17.034 | |
| 34 | 56 | m |  |  |  | 1.1 | 19.0 | 67.414 | 22.378 | 15.652 | 5.101 | 4.272 | 7.195 | |
| 35 | 28 | m |  |  |  | * | 19.0 | 135.462 | 52.555 | 41.418 | 6.234 | 7.094 | 8.066 | |
